# Forecasting and Analyzing the Disease Burden of Aged Population in China, Based on the 2010 Global Burden of Disease Study 

**DOI:** 10.3390/ijerph120707172

**Published:** 2015-06-25

**Authors:** Chengzhen Bao, Mamat Mayila, Zhenhua Ye, Jianbing Wang, Mingjuan Jin, Wenjiong He, Kun Chen

**Affiliations:** 1 School of Public Health, Zhejiang University, Hangzhou 310058, China; E-Mails: baochengzhen@sina.com (C.B.); xabnam316@163.com (M.M.); kimi11089@126.com (Z.Y.); wangjianbing1980@yahoo.com (J.W.); jinmj@zju.edu.cn (M.J.); 2 School of Public Affairs, Zhejiang University, Hangzhou 310058, China; E-Mail: hewenjiong@163.com

**Keywords:** disease burden, aged population, China, Grey model

## Abstract

*Background*: Forecasting the disease burden of the elderly will contribute to make a comprehensive assessment about physical and mental status of the elderly in China and provide a basis for reducing the negative consequences of aging society to a minimum. *Methods*: This study collected data from a public database online provided by Global Burden of Disease Study 2010. Grey model GM (1, 1) was used to forecast all-cause and disease-specific rates of disability adjusted life years (DALYs) in 2015 and 2020. *Results*: After cross-sectional and longitudinal analysis, we found that non-communicable diseases (NCDs) were still the greatest threats in the elderly, followed by injuries. As for 136 predicted causes, more than half of NCDs increased obviously with age, less than a quarter of communicable, material, neonatal, and nutritional disorders or injuries had uptrend. *Conclusions*: The findings display the health condition of the Chinese elderly in the future, which will provide critical information for scientific and sociological researches on preventing and reducing the risks of aging society.

## 1. Introduction

On 28 April 2011, National Bureau of Statistics of China released the first report on the sixth nationwide population census. The report stated that the gross population in China (mainland) had reached 1.3397 billion in 2010, and the proportion of population aged 65 years and older had grown from 7.0% in 2000 to 8.9% in 2010 [1]. According to conservative estimates, by the middle of the 21st century 1/5 to 1/3 of Chinese will be aged 65 years and older, and the proportion of the oldest old, aged 80 years and older, would jump from 1% in 2005 to 6.5%–9% at the same time [2]. Rapid population aging had resulted in increases of some diseases’ morbidity and prevalence rates, resulting in a series of significant scientific and social consequences, especially in medicine and healthcare [3]. On the one hand, medical and health care resources may not be able to meet the ever-increasing needs of elderly patients, which means a relative shortage of medical and health care resources [4]. On the other hand, the relatively stagnant development of geriatrics [5] would be a great barrier to reducing the disease burden of the elderly. To resolve these issues, the most basic and critical thing we should do is to know more about the health condition of the elderly. After that, effective measures (e.g., reasonable resources allocation; establishment and improvement of related discipline, legal and policy system) can then be taken to prevent and reduce the risk of aging society.

The disease burden can be defined as the gap between current health conditions and an ideal situation that everyone lives into old age free of disease and disability [6]. In 1990, the first study of Global Burden of Disease (GBD) quantified the health effects of more than 100 diseases and injuries for eight world regions with a new metric named disability adjusted life year [7,8,9,10], which was an international index for evaluating disease burden and the average level of health. One disability adjusted life year was defined as one lost year of “healthy” life. The disability adjusted life years (DALYs) for a disease or injury contained two parts, that is, the years of life lost due to premature mortality and the years lost due to disability for incident cases of disease or injury [6]. A recent study had shown that in the elderly (55 and older) DALYs for seasonal influenza and Hepatitis B Virus infection would be higher in 2030 than those in 2000 if a static demography was assumed [11]. In addition, it was reported that the DALYs for some chronic diseases such as myocardial infarction would dramatic increase among the elderly in 2025 compared to that in 2004 [12]. All of the above could contribute to assess disease burden among aged population in the future.

The GBD 2010 brought together a great quantity of experts and leaders in epidemiology, economics, statistics and other related areas to study the latest progress of diseases, injuries and risk factors [13]. Based on the findings of GBD 2010, a large number of articles have been published. For China, after comparing all causes and disease-specific rates of DALYs in 1990 and 2020, a health transition in China had been revealed, but not all the data has been analyzed by age [14]. Few studies are available for analyzing the DALYs or estimating that in the future for aged population in China.

The latest disease-specific rates of DALYs that had been released online by GBD 2010 revealed that non-communicable diseases (NCDs) had become the gravest threat in Chinese people aged 60 years and older. The online heat maps showed that in 2010 stroke, ischemic heart disease, chronic obstructive pulmonary disease, lung cancer and liver cancer ranked as the top five among Chinese people aged 60–69 years, diabetes went up to 5th in the age group of 70–79 years, and lower respiratory infections, Alzheimer’s Disease replaced the two cancers mentioned above in adults aged 80 years and older, respectively [15]. GBD 2010 indicated that in 2010, all the top 10 causes among Chinese people aged 60–79 years were NCDs, and as for the top 20 causes, excluding lower respiratory infections, road injury, falls, and self-harm, the others were also NCDs [16].

According to the data published by GBD 2010, this paper forecasts all-cause and disease-specific DALYs (average rate per 100,000) of Chinese adults aged 60–84 years in 2015 and 2020 by a projection model. The findings could help policy makers, medical staffs, researchers, educators and other related social roles to make a comprehensive assessment about physical and mental status of the elderly in China and provided a basis for reducing the negative consequences of aging society to a minimum.

## 2. Methods

### 2.1. Data Collection and Exclusion Criteria

The data used in our study were from an online public database including five cross-sectional datasets from 1990 to 2010, and classification of diseases and injuries was based on a causes list, both of which were provided by GBD 2010. The cause list contained four levels, and each level provided different subsets of overall causes, DALYs (average rate per 100,000) for diseases and injuries in each level were reported. In the overall prediction, all kinds of communicable, material, neonatal, and nutritional disorders was regarded as a whole named group I, all NCDs were in group II, and group III contained all injuries. In separate prediction, this study selected 136 causes (155 causes in total), including 33 communicable, neonatal, and nutritional disorders, 91 NCDs, and 12 injuries in the third level. Some causes that rarely occurred in China (e.g., Chagas disease, African trypanosomiasis, yellow fever) or seldom influenced the health of the elderly (e.g., maternal hemorrhage, maternal sepsis, abortion) were excluded in the separate prediction [17]. For forecasting the disease burden of Chinese adults aged 60 years and older in 2015 and 2020, those younger than 60 years old should be focus on, so that a longitudinal study could be implemented, when analyzing the future disease burden, people aged 60–84 years were divided into five 5-year groups, including 60–64 years, 65–69 years, 70–74 years, 75–79 years and 80–84 years.

### 2.2. Model Selection and Statistical Analysis

Grey system theory is an interdisciplinary scientific theory that is widely used to deal with the systems with several unknown parameters [18]. Compared with general statistic models, the models based on Grey system theory such as GM (1, 1) only need limited data for building and predicting, which could also provide a certain degree of fitting effect to evaluate the reliability [19,20]. Nowadays, GM (1, 1) has been widely used in many fields such as pollutants prediction, traffic flow prediction, yield prediction, and population forecasting [21,22,23,24,25]. In the medical world, Grey models have gained acceptance and been used to forecast mortality and morbidity of various diseases and injuries in China [26]. Considering the characteristics of datasets and reliable prediction results of GM (1, 1) in previous studies, GM (1, 1) type of Grey model is applicable to this study.

As a time series forecasting model, GM (1, 1) could only be used in positive data sequences [20]. Therefore, the data in five cross-sectional datasets should first be arranged in order. For example, for estimating all-cause DALYs (average rate per 100,000) of 60–64 years old people in 2015 and 65–69 years old people in 2020, all-cause DALYs (average rate per 100,000) of five populations (55–59 year old people in 2010, 50–54 year old people in 2005, 45–49 year old people in 2000, 40–44 year old people in 1995, and 35–39 year old people in 1990) should be sorted out and arranged in chronological order. This sequence was defined as original series: $X^{\left(0\right)}=\left\{x^{\left(0\right)}\left(t\right),t=1,2,\cdots ,n\right\}$ [27]. Then, a new series was attained after accumulated generating operation, that is:

(1) $$X^{\left(1\right)}=\left\{x^{\left(1\right)}\left(t\right),t=1,2,\cdots ,n\right\}$$

where $x^{\left(1\right)}\left(t\right)=\overset{t}{\underset{i=1}{\sum }}x^{\left(0\right)}\left(i\right)$

The equation of GM (1, 1) was set up as below:

(2) $$dx^{\left(1\right)}\left(t\right)/dt+ax^{\left(1\right)}\left(t\right)=u$$

where “a” and “u” were constants, which defined as development coefficient and endogenous control coefficient, respectively. If $\hat{a}={\left[a,u\right]}^{T}={\left(B^{T}B\right)}^{-1}B^{T}y_{n}$ , “a” and “u” could be calculated based on least square method where:

$$\text{B}=(\begin{array}{cc} \begin{array}{c} -\left[x^{\left(1\right)}\left(2\right)+x^{\left(1\right)}\left(1\right)\right]/2\\ -\left[x^{\left(1\right)}\left(3\right)+x^{\left(1\right)}\left(2\right)\right]/2 \end{array} & \begin{array}{c} 1\\ 1 \end{array}\\ \begin{array}{c} \vdots \\ -\left[x^{\left(1\right)}\left(n\right)+x^{\left(1\right)}\left(n-1\right)\right]/2 \end{array} & \begin{array}{c} \vdots \\ 1 \end{array} \end{array})\text{, }y_{n}={\left(\begin{array}{cc} \begin{array}{cc} x^{\left(0\right)}\left(2\right), & x^{\left(0\right)}\left(3\right) \end{array}, & \begin{array}{cc} \cdots , & x^{\left(0\right)}\left(n\right) \end{array} \end{array}\right)}^{T}$$

Based on the following equation, the predicted series could be attained:

(3) $${X_{p}}^{\left(0\right)}=\left\{\left(e^{-a}-1\right)\left[x^{\left(0\right)}\left(1\right)-u/a\right]e^{-at},\;t=1,2,\cdots ,n\right\}$$

According to the mathematical principles of GM (1, 1) [20], we compiled a series of SAS programs to forecast DALYs (average rate per 100,000) and then evaluate the reliability by computing the variance ratio called C (the ratio between the variance of residual error and the variance of original data), if C ≤ 0.35 then the fitting quality was good, if 0.35 < C ≤ 0.5 then it was eligible, else if C > 0.65 then it was the worst grade [28,29,30,31].

Paired samples t-test was used to analyze whether the difference between the predicted and reported groups was significant or not. All-cause DALYs (average rate per 100,000) of 11 age groups in 2010 were regarded as controls, that is, reported group. 22 GM (1, 1) were built with all-cause DALYs (average rate per 100,000) of three or four cross-sectional dataset (from 1990 to 2000/2005). Based on the number of data in original series, 22 predicted DALYs (average rate per 100,000) of 11 age groups in 2010 were classified into two predicted groups. On account that the d-values of reported and predicted data fitted normal distribution, we used paired samples *t*-test to analyze the consistency between reported data and one group of predicted data separately.

## 3. Results

### 3.1. Comparing Predicted Data with Reported Data

**Figure 1:**
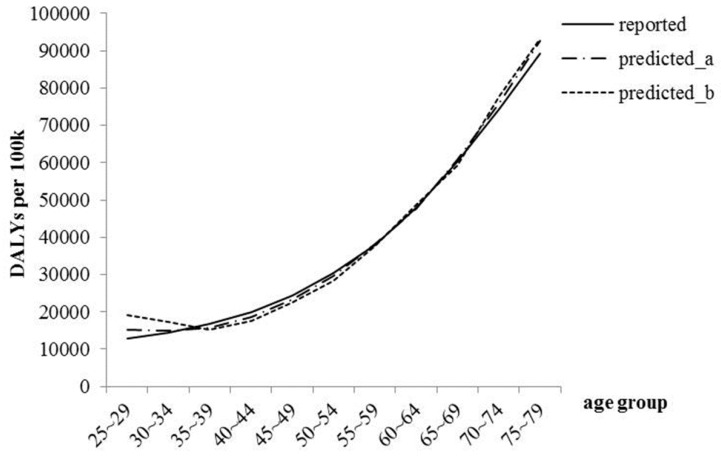
The disability adjusted life years (DALYs) of Chinese people for all kinds of diseases and injuries in 2010. Reported: composed of 11 datasets, which were published online. Predicted_a: composed of 11 datasets in predicted group a, which were respectively predicted by 11 original series that including four cross-sectional datasets (from 1990 to 2005). Predicted_b: composing of 11 datasets in predicted group b, which were respectively predicted by 11 original series that included three cross-sectional datasets (from 1999 to 2005).

The result of paired samples t-test indicated that the differences were non-significant when the reported group was compared with the predicted group a (*p* = 0.41) and b (*p* = 0.39) separately. Figure 1 presents the difference between predicted and reported data. Obviously, the predicted data were quite close to the reported data.

### 3.2. Overall Forecasting and Analyzing the Disease Burden

**Figure 2:**
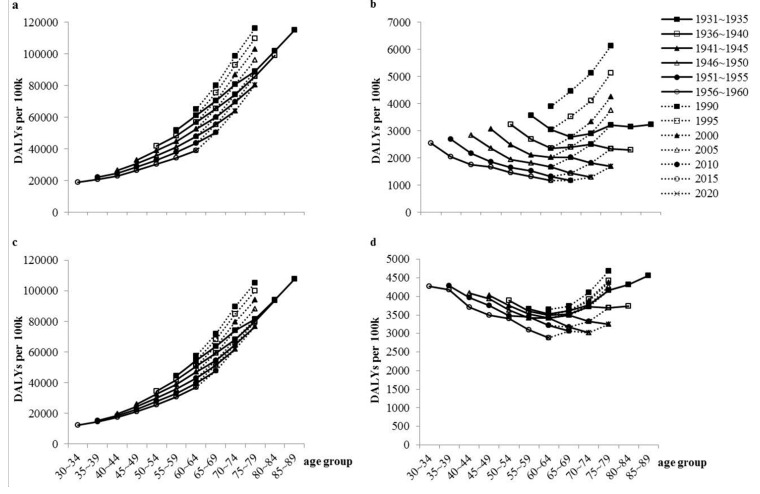
The disability adjusted life years (DALYs) of Chinese people: longitudinal and cross-sectional analysis between 1990 and 2020. (**a**) The average DALYs (per 100,000) for all kinds of diseases and injuries; (**b**) The average DALYs (per 100,000) for communicable, maternal, neonatal, and nutritional disorders diseases; (**c**) The average DALYs (per 100,000) for non-communicable diseases; (**d**) The average DALYs (per 100,000) for injuries.

After cross-sectional and longitudinal analysis, we determined the tendencies of all-cause and disease-specific DALYs (average rate per 100,000). Figure 2a shows that in each birth cohort all-cause DALYs (average rate per 100,000) increases with age. In addition, the earlier-born cohort in each age group always had higher DALYs (average rate per 100,000). Through cross-sectional analysis (dotted line), we found that as time goes on all-cause DALYs (average rate per 100,000) decreased in each age group.

Figure 2b shows similar characteristics to those presented in Figure 2a, when comparing the changes of DALYs (average rate per 100,000) in different survey years. However the tendencies of birth cohorts were inconsistent with that in Figure 2a, as almost all cohorts were declining steady (exc., 1931–1935). We conjectured that in the elderly the harmful effects of group I would not be greater and greater with age until they became the oldest old, which may relate to its higher rate of fatalities than in the younger elderly.

The findings of the longitudinal and cross-sectional analysis in Figure 2c were almost the same as those in Figure 2a, and the only difference was that the object of study was replaced by group II. Obviously, group II played an important role in the change of all-cause DALYs (average rate per 100,000). Figure 2b,d had quite a few similarities in the curve characteristics, and the main difference was that group III affected the health of the elderly, especially the oldest old, more dramatically than group I.

### 3.3. Separate fore Casting and Analyzing the Disease Burden

Tables S1 to S4 provide overviews of the results of the separate prediction, and DALYs for 136 causes in 2015, 2020 are shown, respectively. When the top 10 causes list of each age group in 2010 is compared with those in 2015 and 2020, the categories of disease and injury were almost the same. Slight differences among the three cross-sections could be found as follows: osteoarthritis that ranked 14th in the elderly aged 60–64 and 65–69 in 2010 would raise up to 10th in the former age group in 2015, and it might bring more DALYs (average rate per 100,000) and ran up to 6th and 10th separately in these two age groups after five years. In addition, esophageal cancer (rank 13th in 2010) would be in the top 10 causes list in the elderly aged 75–79 by 2020.

To compare the reported DALYs (average rate per 100,000) in 2010 with the predicted data in 2015 and 2020, we selected 136 predicted causes from all reported causes and re-ranked them by DALYs (average rate per 100,000). Then we found that in each age group, the trends of DALYs (average rate per 100,000) for nearly or more than a half causes were the same as rank. To be specific, some causes might descend continuously (e.g., diarrheal diseases, lower respiratory infections, and meningitis), and some would have uptrends (e.g., alcohol use disorders, nasopharynx cancer, and breast cancer). At the same time, there was a fraction of causes whose trends of DALYs (average rate per 100,000) were opposite to rank (e.g., leishmaniasis, dengue, and iodine deficiency).

Based on the reported and predicted data of 136 predicted causes, we did a longitudinal analysis of DALYs (average rate per 100,000) as before. We found that in the elderly more than half of causes that belonged to group I or III decreased with age more or less, and less than a quarter of which had uptrend (e.g., lower respiratory infections, trachoma, varicella, falls, and adverse effects of medical treatment). Whereas, more than half of NCDs increased obviously with age, including some causes (e.g., cerebrovascular disease, trachea, bronchus and lung cancers, and ischemic heart disease) that always could be seen in the top five lists, and less than one third of which had downtrends. 

## 4. Discussion

### 4.1. Aging Society and Population Health

Incidence, prevalence and death rate, those indicators used previously for the measures of ill-health cannot indicate loss of one’s overall health, and it is reasonable to introduce indicators such as DALYs for evaluating the level of population health. Therefore, the prediction of DALYs plays an important role in estimating health status in the future. 

Population aging will bring great threats to the general level of health. The latest study reconfirmed that the body function, self-care capacity and general physical health condition gradually decreased with age [32]. According to the report published by China Research Center on Ageing, there were 202 million elderly people living in China in 2013, and nearly half of which had NCDs, more than 18(%) of which had disabilities [33]. In this study, the DALYs (average rate per 100,000) are decreasing with time in each age group as Figure 2 shown, but a larger proportion of the elderly is likely to bring a larger DALYs in the future. For improving health status of the elderly, researchers, physicians, nurses, public health staff, and other medical workers should pay more attention to those disease and injuries that DALYs for which will increase with age obviously, especially after 60 years of age.

Population aging will place more demands on advanced geriatric medicine and nursing. The treatments for the elderly cannot not be similar to those for the young in many situations [34]. In 2006, a joint international initiative between Peking Union Medical College and Johns Hopkins University School of Medicine was initiated, which aimed at promoting quality geriatrics care, education, and aging research across China [35]. However, advanced geriatric medicine and nursing was still in short supply. The development of elderly-friendly medical devices and nursing-care equipment was considered essential to reduce the demand of labor resource [36]. Rational senior wellness programs could bring positive health outcomes [37]. Based on our results, the kernel for developing geriatric medicine and nursing will be clearer than before, that is, those diseases and injuries ranked in top 10 or 20 in 2020 should be taken into consideration firstly, which should be beneficial to reduce the risks of an aging society.

### 4.2. Aging Society and Health Economics

As an indicator of health economics, disease burden is always used to evaluate cost-effect, cost-efficiency and cost-efficiency. Through forecasting all-cause and disease-specific DALYs (average rate per 100,000), overall and part economic evaluation can be implemented, which will contribute to improve disease prevention, treatment and control technology. In addition, for solving other health economics issues such as short-term and long-term planning of health resources allocation, the present and future disease burden should be taken as reference. 

Population aging will be a great challenge for making fairer and more effective allocations of health resources. Facing the growing demands of the elderly, health systems should cost more to cover chronic diseases and disabilities [38] and improve primary care [39], but recent studies show that equality and effectiveness of public health services for patients with chronic diseases were far from satisfying in China [40]. In China, poorly skilled doctors, unsatisfactory services and low-quality medical equipment were still provided for the elderly in community-based primary health care [41]. Therefore, with the coming of aging society, we should find a way to make reasonable allocation of health resources. A latest study referred to a global threshold, that is, the minimum healthcare services that could reduce the global DALYs to the minimum [42]. By knowing more about all-cause and disease-specific rates of DALYs in the elderly, we can have substantial cost benefits in an aging society and even find out a Chinese threshold to increase availability and proportionality of health resources in the future.

### 4.3. Aging Society and Health Policy

Population aging will result in a series of public policy responses. In the field of medicine, this mainly affects health insurance policy and disease prevention policy. Disease burden measure is the gist to establish relevant policies, and a rational and quantitative prediction of disease burden will optimize long-term effect.

In China, the “structural deficiency” of the health care system is still serious at present. On the one hand, there are defects in the health insurance system, such as urban employee’s basic medical insurance being directly tied to employment, which had adverse effects on the availability of health care [43]. On the other hand, medical treatment always draws more attention than disease prevention, so that it is still relatively passive in the aspect of improving health conditions. Population aging will result in variances of the disease burden and then the medical service needs will be different. For health policy, long-term effects should be taken into consideration. The higher the disease burden, the greater the medical demands and medical costs may be. Therefore, when improving the health insurance system in an aging society, we should take full account of the needs of the elderly and properly increase the proportion of expenditures on the elderly for diseases and injuries with great disease burden. In addition, a series of new disease prevention policies such as higher prices of and restriction for use of tobacco and alcohol, which can change general behavioral patterns, should be established [44,45]. For diseases and injuries ranked in the top 10 lists that we have mentioned, effective health education and periodic physical examinations could be carried out.

### 4.4. Strengths and Limitations

This study has several strengths, including its use of the latest international data to qualitatively and quantitatively assess health conditions among aged Chinese people from a multi-dimensional point of view. Some limitations must however be acknowledged. Firstly, even though other studies have confirmed that the prediction accuracy was hardly influenced by the number of training samples [46], the longer the forecast period was, the greater the uncertainty would be. Secondly, we could not separate the data into urban and rural groups, but the major features and needs of medical and health services might be quite different in these two groups [47,48]. Thirdly, considering the length of the article, we have not done a gender-specific project.

## 5. Conclusions

This study collected official data at five survey years. Based on these data, we quantitatively estimated the coming disease burden by building large numbers of predictive models. Through computing the variance ratio in each model and analyzing significant differences between reported and predicted groups, we made the degree of fitting of the model clear. The results showed that all-cause DALYs (average rate per 100,000) of the elderly in China obviously increase with age, which was mainly affected by NCDs. In 2010, excluding people aged 80 years and older, the majority of causes in the top 10 causes list were NCDs, and this list would be nearly unchanged ten years later. In each age group, as time goes on, the trends of DALYs for nearly or more than half of causes were the same as their ranking. More than half of causes that belonged to group I or III decreased with age more or less, and less than a quarter of which had uptrends. More than half of causes in group II increased obviously with age, including some causes that always could be seen in the top five lists. All conclusions in our study could reveal the health condition of the Chinese elderly in the future from a new angle, and then provide critical information for scientific and sociological research on preventing and reducing the risks of an aging society.

## Supplementary Files

Supplementary File 1

## Abbreviations

DALYs, Disability adjusted life years; GBD, Global Burden of Disease; NCDs, Non-communicable diseases.

## References

[1] Peng X.-Z. (2011). China’s demographic history and future challenges. Science.

[2] Mai Y.-H., Peng X.-J., Chen W. (2013). How fast is the population ageing in China?. Asian Popul. Stud..

[3] Koch S. (2010). Healthy ageing supported by technology––A cross––Disciplinary research challenge. Inform. Health Soc. Care.

[4] O’Brien T. (2013). The impact of an aging population on palliative care. J. Pain Palliat. Care Pharmacother..

[5] Chen Z., Yu J., Song Y.-T., Chui D.-H. (2010). Aging Beijing: Challenges and strategies of health care for the elderly. Ageing Res. Rev..

[6] World Health Organization (WHO) The Global Burden of Disease: 2004 Update..

[7] World Bank (1993). World Development Report 1993: Investing in Health.

[8] Murray C.J.L., Lopez A.D. (1996). Evidence––Based health policy––Lessons from the global burden of disease study. Science.

[9] Murray C.J.L., Lopez A.D. (1996). The Global Burden of Disease: A Comprehensive Assessment of Mortality and Disability from Diseases, Injuries and Risk Factors in 1990 and Projected to 2020.

[10] Murray C.J.L., Murray C.J.L., Lopez A.D. (1996). Rethinking DALYs. The Global Burden of Disease.

[11] McDonald S.A., van Lier A., Plass D., Kretzschmar M.E.E. (2012). The impact of demographic change on the estimated future burden of infectious diseases: Examples from hepatitis B and seasonal influenza in the Netherlands. BMC Public Health.

[12] Terschuren C., Mekel O.C.L., Samson R., Classen T.K.D., Hornberg C., Fehr R. (2009). Health status of “Ruhr-City” in 2025––Predicted disease burden for the metropolitan Ruhr area in North Rhine-Westphalia. Eur. J. Public Health.

[13] (2013). Institute for Health Metrics and Evaluation (IHME). The Global Burden of Disease: Generating Evidence, Guiding Policy.

[14] Yang G.H., Wang Y., Zeng Y.X., Gao G.F., Liang X.F., Zhou M.G., Wan X., Yu S.C., Jiang Y.H., Naghavi M. (2013). Rapid health transition in China, 1990–2010: Findings from the global burden of disease study 2010. Lancet.

[15] Institute for Health Metrics and Evaluation (IHME) GBD Heatmap.

[16] Institute for Health Metrics and Evaluation (IHME) GBD Database.

[17] Murray C.J.L., Ezzati M., Flaxman A.D., Lim S., Lozano R., Michaud C., Naghavi M., Salomon J.A., Shibuya K., Vos T. (2012). GBD 2010: Design, definitions, and metrics. Lancet.

[18] Lin Y., Liu S.-F. A historical introduction to grey systems theory. Proceedings of the 2004 IEEE International Conference on Systems, Man, and Cybernetics, Hague, The Netherlands.

[19] Deng J.-L. (1982). Control problems of grey system. Syst. Control. Lett..

[20] Deng J.-L. (1989). Introduction to grey system theory. J. Grey Syst..

[21] Huang L., Gao X., Liu M., Du G., Guo J.-S. (2013). SO_2_ in atmosphere predicted with improved error GM (1, 1) model-based on optimization of initial condition in Chongqing, China. Asian J. Chem..

[22] Pai T.-Y., Ho C.-L., Chen S.-W., Lo H.-M., Sung P.-J., Lin S.-W., Lai W.-J., Tseng S.-C., Ciou S.-P., Kuo J.-L. (2011). Using seven types of GM (1, 1) model to forecast hourly particulate matter concentration in Banciao city of Taiwan. Water Air Soil Pollut..

[23] Mao S.-H., Chen Y., Xiao X.-P. (2012). City traffic flow prediction based on improved GM (1, 1) model. J. Grey Syst..

[24] Zhou W., He J.-M. (2013). Generalized GM (1, 1) model and its application in forecasting of fuel production. Appl. Math. Model..

[25] Chen Y.-M., Zhu M.-L. (2011). Population forecasting of Zhan-Li Dong-Ethnic village based on GM (1, 1) model. J. Grey Syst..

[26] Wu W., Guan P., Guo J.-Q., Zhou B.-S. (2008). Comparison of GM (1, 1) gray model and ARIMA model in forecasting the incidence of hemorrhagic fever with renal syndrome. J. China Med. Univ..

[27] Deng J.-L. (1987). Basic Methods of Grey System.

[28] Deng J.-L. (2002). Grey Theoretical Basis.

[29] Deng J.-L. (2002). Grey Prediction and Grey Decision-making.

[30] Kong C., Liu Y.-F., Shen X.-L. (2008). Improvement on SAS programs for the grey forecasting model. Chinese J. Health Stat..

[31] Wang Y.-X., Du Q.-Y., Ren F., Liang S., Lin D.-N., Tian Q., Chen Y., Li J.-J. (2014). Spatio-temporal variation and prediction of Ischemic Heart Disease hospitalizations in Shenzhen, China. Int. J. Environ. Res. Public Health.

[32] Chao J.-Q., Li Y.-Y., Xu H., Yu Q., Wang Y.-M., Liu P. (2013). Health status and associated factors among the community-dwelling elderly in China. Arch. Gerontol. Geriatr..

[33] Wu Y.-S., Dang J.-W. (2013). China Report of the Development on Aging Cause.

[34] Samuel M.-J. (2013). American geriatrics society identifies five things that healthcare providers and patients should question. J. Am. Geriatr. Soc..

[35] Leng S.-X., Tian X.-P., Liu X.-H., Lazarus G., Bellantoni M., Greenough W., Fried L.-P., Shen T., Durso S.C. (2010). An international model for geriatrics program development in China: The Johns Hopkins-Peking Union Medical College experience. J. Am. Geriatr. Soc..

[36] Arai H., Ouchi Y., Yokode M., Ito H., Uematsu H., Eto F., Oshima S., Ota K., Saito Y., Sasaki H. (2012). Toward the realization of a better aged society: Messages from gerontology and geriatrics. Geriatr. Gerontol. Int..

[37] Coberley C., Rula E.Y., Pope J.E. (2011). Effectiveness of health and wellness initiatives for seniors. Popul. Health Manag..

[38] Liu S., Griffiths S.M. (2011). From economic development to public health improvement: China faces equity challenges. Public Health.

[39] Bodenheimer T., Pham H.H. (2010). Primary care: Current problems and proposed solutions. Health Aff..

[40] Tian M.-M., Feng D., Chen X., Chen Y.-C., Sun X., Xiang Y.-X., Yuan F., Feng Z.-C. (2013). China’s rural public health system performance: A cross-sectional study. PLoS ONE.

[41] Niu T.-H., Meng Q.-Y., Meng X.-Z., Li X.-M., Zhai Q., Li X.-Y. (2010). The analysis on the satisfactory degree of community health service and its influencing factors among the rural elders. Chin. J. Health Stat..

[42] Drake T. (2014). Priority setting in global health: Towards a minimum DALY value. Health Econ..

[43] Hou J.W., Li K.W. (2011). The aging of the Chinese population and the cost of health care. Soc. Sci. J..

[44] Huang C., Yu H., Koplan J.P. (2014). Can China diminish its burden of non-communicable diseases and injuries by promoting health in its policies, practices, and incentives?. Lancet.

[45] Bloom D.E., Chatterji S., Kowal P., Lloyd-Sherlock P., Mckee M., Rechel B., Rosenberg L., Smith J.P. (2015). Macroeconomic implications of population ageing and selected policy responses. Lancet.

[46] Fu J.-H., Tong J., Wang Q., Wang Z.-Y. (2011). A data prediction method under small sample condition by combining neural network and grey system methods. Proc. SPIE.

[47] Wang H.-H., Huang S.-M., Zhang L.-X., Rozelle S., Yan Y.-Y. (2010). A comparison of rural and urban healthcare consumption and health insurance. China Agric. Econ. Rev..

[48] Yu P.-L., Song X.-W., Shi J. (2012). Frailty and survival of older Chinese adults in urban and rural areas: Results from the Beijing longitudinal study of aging. Arch. Gerontol. Geriatr..

