# Supplementary material for: Forecasting and Analyzing the Disease Burden of Aged Population in China, Based on the 2010 Global Burden of Disease Study
Source: Int J Environ Res Public Health. 2015 Jun 25;12(7):7172–84. doi: 10.3390/ijerph120707172 (PMC4515648; doi:10.3390/ijerph120707172)
Supplement: Supplementary File 1 [file ijerph-12-07172-s001.pdf]

Forecasting and Analyzing the Disease Burden of Aged Population in China, Based on the Global Burden of Disease Study 2010

Table S1. Disability adjusted life years (DALYs) for 33 communicable, neonatal, and nutritional disorders.

Short Cause Names	Average DALYs per 100,000 (Rank in 136 Causes)									
	60–64 Years		65–69 Years		70–74 Years		75–79 Years		80–84 Years	
	2015	2020	2015	2020	2015	2020	2015	2020	2015	2020
TB	167 (43)	127 (54)	194 (44)	126 (56)	279(37)	144 (52)	292 (36)	221 (46)	285 (39)	221 (47)
HIV	110 # (60)	313 # (31)	82 # (70)	133 # (55)	61 # (82)	98 # (65)	− (−)	− (−)	− (−)	− (−)
Diarrhea	56 (76)	51 (79)	59 (78)	50 (83)	60 (83)	52 (85)	72 (83)	51 (89)	85 (77)	63 (86)
Typhoid	13 (111)	14 (108)	10 (113)	10 (111)	9 (115)	8 (112)	14 # (109)	8 (115)	18 (107)	14 # (106)
LRI	254 (32)	173 (43)	410 (26)	289 (34)	752 (21)	490 (27)	1386 (14)	988 (18)	2286 (10)	1967 (13)
URI	7 (115)	10 * (111)	4 (122)	6 (118)	3 (130)	3 (123)	4 * (124)	2 (129)	11 * (115)	4 * (122)
Otitis	50 (81)	41 (87)	58 * (79)	48 (85)	79 ## (75)	57 * (81)	124 (59)	84 ## (73)	180 (52)	148 (60)
Meningitis	38 (90)	26 (99)	50 (87)	31 (96)	75 * (79)	44 (93)	103 (67)	71 * (79)	124 ## (62)	102 (66)
Enceph	2 (128)	2 (129)	3 (129)	2 (129)	3 (129)	2 (127)	3 (126)	2 (126)	4 (126)	3 (125)
Tetanus	5 (123)	3 (125)	4 (121)	4 (122)	3 (125)	3 (122)	2 (129)	2 (127)	5 (123)	1 (129)
Varicella	9 (112)	7 * (114)	11 (112)	11 (110)	13 (110)	14 (107)	13 (110)	15 (107)	14 (111)	15 (105)
Malaria	1 (133)	0 * (133)	1 * (132)	0 (133)	1 ## (132)	1 * (130)	1 # (131)	1 ## (131)	1 ## (131)	1 # (131)
Leish	3 (125)	3 (126)	3 (126)	2 (125)	3 (128)	2 (128)	3 (127)	2 (128)	3 (128)	2 (128)
Schisto	45 (84)	45 (82)	52 (85)	50 (84)	56 * (84)	55 (83)	57 ## (89)	56 * (85)	56(90)	55 ## (89)
Cysticer	6 * (119)	5 * (121)	6 * (118)	5 * (120)	6 * (119)	5 * (119)	6 *(118)	5 * (120)	6 * (121)	5 *(119)
Echino	6 * (117)	6 (117)	7 * (116)	7 * (115)	8 (117)	7 * (114)	8 (114)	9 (111)	7 ## (117)	9 (111)
Trachoma	7 (116)	7 (115)	9 (115)	8 (112)	13 (109)	11 (110)	20 (103)	17 (104)	24 (101)	27 (97)
Dengue	2 (129)	2 (128)	3 ## (127)	2 (126)	3 (126)	3 ## (125)	3 ## (128)	4 (123)	3 ## (129)	3 ## (126)
Rabies	6 # (118)	6 # (118)	6 * (117)	6 # (117)	5 * (120)	6 * (118)	5 (122)	5 * (118)	4 (125)	5 (120)
Nematode	14 (109)	8 (113)	14 (108)	8 (113)	14 (108)	8 (113)	14 (107)	8 (114)	15 (110)	8 (113)
FBT	120 (53)	114 (57)	110 (61)	108 (61)	96 (67)	96 (66)	84 (76)	81 (75)	75 (84)	70 (81)
Oth NTD	23 (103)	22 (101)	12 (111)	17 (108)	9 (116)	7 (115)	6 (119)	5 (117)	7 (119)	3 (123)
Preterm	31 (98)	32 (96)	30 (100)	30 (97)	28 (101)	29 (101)	26 (99)	27 (98)	24 (102)	24 (98)
N Enceph	46 (83)	46 (81)	40 (96)	40 (90)	33 (96)	34 (96)	26 (97)	27 (97)	21 (103)	21 (101)

Table S1. *Cont.*

Short Cause Names	Average DALYs per 100,000 (Rank in 136 causes)									
	60–64 Years		65–69 Years		60–64 Years		75–79years		60–64 Years	
	2015	2020	2015	2020	2015	2020	2015	2020	2015	2020
PEM	8 (113)	5 (122)	13 * (109)	7 (114)	18 (106)	11 * (108)	34 # (94)	17 (105)	69 * (87)	36 #(94)
Iodine	20 (104)	19 (103)	20 (106)	19 (106)	20 (105)	19 (105)	19 (104)	18 (103)	19 (105)	18 (103)
Vit A	0 (134)	0 (134)	0 (134)	0 (134)	0 (133)	0 (134)	0 (132)	0 (132)	0 (132)	0 (132)
Iron	56 (77)	49 (80)	60 (77)	47 (86)	52 (87)	52 (86)	62 (86)	43 (92)	89 # (74)	55 (90)
Oth Nutr	2 (127)	2 (130)	3 * (124)	2 (127)	4 (123)	3 * (124)	6 ## (115)	3 (124)	12 * (114)	6 ## (116)
STD	2 (131)	2 (127)	3 (128)	1 (131)	2 (131)	1 (129)	2 (130)	1 (130)	1 (130)	1 (130)
Hep	293 * (30)	313 (32)	293 # (34)	345 * (30)	296 # (35)	337 # (33)	255 # (39)	342 #(35)	210 (48)	286 # (39)
Leprosy	0 (136)	0 (136)	0 (135)	0 (136)	0 (135)	0 (135)	0 (134)	0 (134)	0 (134)	0 (134)
Oth Inf	68 (70)	62 * (73)	90 (66)	79 (70)	105 (63)	109 (63)	119 (61)	131 (61)	141 (59)	151 (56)

* $0.35 < C \leq 0.5$, the fitting effect is eligible; # $0.5 < C \leq 0.65$, the fitting effect is below standard, but not the worst; ## $C > 0.65$, the fitting effect is fairly unsatisfactory. The average DALYs (per 100,000) are rounded to the nearest integers in the table, and the rank lists are got according to the original data. The full cause names are presented in Table S5.

Table S2. Disability adjusted life years (DALYs) for 28 neoplasms.

Short Cause Names	Average DALYs per 100,000 (Rank in 136 Causes)									
	60–64 Years		65–69 Years		70–74 Years		75–79 Years		80–84 Years	
	2015	2020	2015	2020	2015	2020	2015	2020	2015	2020
Esophagus	1362 (9)	1584 (9)	1764 (9)	1990 (9)	1829 * (10)	2341 (9)	1702 * (11)	2135 * (10)	1465 ## (15)	1776 * (16)
Stomach	1694 (7)	1352 (10)	2407 (7)	2138 (8)	2934 (5)	3003 (5)	3075 * (7)	3479 (5)	2770 ## (8)	3384 * (7)
Liver	2532 (5)	2807 (5)	2602 (6)	2969 (5)	2524 (8)	2858 (7)	2173 * (9)	2620 (9)	1936 (13)	2117 * (10)
Larynx	114 (58)	157 (47)	141 (50)	175 (47)	144 (51)	194 (44)	139 (55)	174 (51)	111 # (67)	150 (57)
Lung	3628 (2)	3915 (2)	4796 (3)	5630 (2)	5643 (4)	6993 (3)	5912 (4)	7616 (4)	5324 (4)	7296 (4)
Breast	432 (23)	595 (19)	321 # (28)	495 (24)	255 ## (39)	325 # (34)	216 (47)	244 ## (43)	208 (49)	204 (50)
Cervix	158 (46)	262 (35)	130 (53)	184 (44)	118 ## (59)	133 (55)	105 * (65)	114 ## (63)	111 * (66)	97 * (69)

Table S2. *Cont.*

**Short Cause Names**	**Average DALYs per 100,000 (Rank in 136 Causes)**									
	**60–64 Years**		**65–69 Years**		**70–74 Years**		**75–79 Years**		**80–84 Years**	
	**2015**	**2020**	**2015**	**2020**	**2015**	**2020**	**2015**	**2020**	**2015**	**2020**
Uterus	105 (61)	143 (51)	84 (69)	154 (53)	79 (76)	101 (64)	65 (84)	87 (70)	60 $^{\#}$ (88)	66 (83)
Prostate	14 $^{\#}$ (108)	11 (109)	53 (84)	28 $^{\#}$ (99)	135 (54)	124 (59)	220 (46)	305 (36)	291 (36)	441 (33)
Colorectal	813 (15)	746 (15)	1079 (13)	1075 (15)	1373 (12)	1401 (12)	1586 (13)	1750 (11)	1677 (14)	1947 (14)
Mouth	111 (59)	170 (45)	105 * (63)	155 (52)	113 (60)	130 * (56)	115 * (63)	134 (59)	102 $^{\#}$ (71)	133 * (61)
Naso	223 * (35)	274 (34)	213 (41)	246 * (39)	212 $^{\#\#}$ (43)	223 (42)	170 $^{\#\#}$ (51)	220 $^{\#\#}$ (47)	125 $^{\#}$ (61)	164 $^{\#\#}$ (54)
Oth Pharynx	42 (86)	58 (74)	37 * (97)	59 (77)	28 (100)	44 * (91)	25 (102)	27 (96)	28 * (99)	23 (100)
Gallbladder	167 (44)	180 (42)	224 (39)	256 (38)	284 (36)	322 (35)	354 (33)	386 (33)	359 (34)	466 (32)
Pancreas	430 (25)	504 (22)	544 (23)	685 (21)	601 (24)	794 (22)	608 (27)	795 (23)	545 (31)	737 (26)
Melanoma	37 (93)	42 (85)	42 (91)	54 (79)	48 (91)	58 (80)	60 (87)	66 (82)	70 (86)	81 (77)
NMSC	20 (105)	17 (104)	23 (103)	27 (101)	34 (94)	29 (100)	40 (93)	44 (91)	50 (92)	50 (91)
Ovary	166 * (45)	252 (36)	140 $^{\#}$(52)	198 * (42)	111 * (62)	144 $^{\#}$ (53)	103 $^{\#}$ (66)	103 * (67)	87 * (76)	94 $^{\#}$ (70)
Testis	1 (132)	1 (131)	1 (131)	1 (132)	3 * (127)	1 (131)	5 $^{\#\#}$ (121)	4 * (122)	3 $^{\#\#}$ (127)	6 $^{\#\#}$ (117)
Kidney	252 (33)	340 (29)	273 (35)	406 (27)	300 (34)	396 (29)	303 (35)	408 (32)	288 (37)	387 (35)
Bladder	85 (68)	78 (67)	128 (54)	122 (58)	193 (46)	178 (48)	286 (37)	262 (39)	375 (33)	391 (34)
Brain	274 (31)	315 (30)	312 (30)	308 (32)	333 (31)	342 (32)	316 $^{\#\#}$ (34)	353 (34)	222 (45)	317 $^{\#\#}$ (37)
Thyroid	53 (79)	57 (75)	60 (76)	74 (72)	69 (81)	77 (75)	81 (77)	86 (71)	80 (79)	99 (68)
Hodgkin's	4 $^{\#\#}$ (124)	3 (123)	4 $^{\#}$ (120)	4 $^{\#\#}$ (123)	5 (121)	4 $^{\#}$ (120)	6 $^{\#}$ (117)	5 (119)	6 (120)	6 $^{\#}$ (118)
Lymphoma	153 (47)	147 (49)	203 (42)	202 (41)	215 (42)	267 (39)	222 (44)	261 (40)	204 (50)	253 (43)
Myeloma	64 (74)	54 (77)	86 (68)	98 (64)	99 (65)	126 (58)	99 (69)	134 (60)	78 * (81)	122 (63)
Leukemia	181 $^{\#}$ (41)	138 * (52)	228 $^{\#\#}$ (38)	181 $^{\#}$ (45)	251(40)	243 $^{\#\#}$ (40)	231 * (41)	273(37)	214 $^{\#}$ (46)	239 * (45)
Oth Neoplasm	620 * (18)	631 * (17)	727 $^{\#}$ (18)	709 * (20)	757 $^{\#\#}$ (20)	795 $^{\#}$ (21)	684 $^{\#\#}$ (24)	782 $^{\#\#}$ (24)	644 $^{\#\#}$ (26)	647 $^{\#\#}$ (29)

* $0.35 < C \leq 0.5$, the fitting effect is eligible; $^{\#}$ $0.5 < C \leq 0.65$, the fitting effect is below standard, but not the worst; $^{\#\#}$ $C > 0.65$, the fitting effect is fairly unsatisfactory. The average DALYs (per 100,000) are rounded to the nearest integers in the table, and the rank lists are got according to the original data. The full cause names are presented in Table S5.

Table S3. Disability adjusted life years (DALYs) for 63 non-communicable diseases excluding neoplasms.

Short Cause Names	Average DALYs per 100,000 (Rank in 136 Causes)									
	60–64 Years		65–69 Years		70–74 Years		75–79 Years		80–84 Years	
	2015	2020	2015	2020	2015	2020	2015	2020	2015	2020
Rheum HD	204 (37)	118 (55)	248 (37)	175 (46)	314 (32)	207 (43)	369 (32)	265 (38)	429 (32)	313 (38)
IHD	3454 (3)	3373 (3)	5140 (2)	4718 (3)	7184 (2)	7020 (2)	9751 (3)	9673 (2)	12960 (3)	12895 (3)
Stroke	6385 (1)	6910 (1)	9395 (1)	9021 (1)	13155 (1)	12662 (1)	17985 (1)	16930 (1)	23183 (1)	22519 (1)
HTN Heart	530 (20)	557 (21)	836 (17)	799 (18)	1230 (14)	1215 (15)	1795 (10)	1683 (12)	2379 (9)	2401 (9)
CMP	118 * (56)	104 * (60)	157 * (48)	126 * (57)	199 (45)	173 * (49)	225 (43)	226 (44)	249 (43)	247 (44)
AFib	102 (63)	103 (61)	172 (46)	165 (48)	276 (38)	283 (38)	427 (31)	445 (31)	606 (29)	678 (27)
AA	186 (40)	184 (41)	300 (33)	261 (37)	500 (28)	429 (28)	743 (23)	734 (25)	849 (21)	1078 (19)
PVD	37 (92)	69 (71)	57 (80)	65 (76)	86 (73)	94 (68)	136 (57)	138 (57)	197 (51)	220 (48)
Endocarditis	25 * (101)	23 * (100)	42 (93)	20 * (105)	55 (85)	41 (94)	59 (88)	60 (83)	81 (78)	63 (85)
Oth Circ	432 (24)	607 (18)	556 (22)	721 (19)	770 (19)	1008 (18)	1030 (18)	1463 (15)	1386 (16)	2004 (11)
COPD	2238 (6)	1645 (8)	4092 (4)	2827 (6)	6652 (3)	5354 (4)	10626 (2)	8451 (3)	14010 (2)	13247 (2)
P-coniosis	32 (97)	29 (97)	44 (89)	43 (87)	72 (80)	56 (82)	86 (75)	93 (69)	92 * (72)	102 (67)
Asthma	83 (69)	76 (68)	105 (62)	86 (68)	159 (49)	111 (61)	239 (40)	179 (50)	319 (35)	278 (41)
Int Lung	38 (91)	36 (90)	53 (83)	54 (80)	77 (78)	75 (76)	118 (62)	107 (65)	143 (58)	169 (53)
Oth Resp	194 (39)	198 (39)	259 (36)	265 (36)	376 (30)	378 (30)	548 (29)	569 (29)	769 (23)	838 (21)
Cirrhosis-HepB	314 * (28)	310 (33)	316 * (29)	317 * (31)	302 (33)	291 * (36)	273 (38)	260 (41)	244 (44)	222 (46)
Cirrhosis-HepC	148 (48)	148 (48)	155 * (49)	155 (51)	153 (50)	147 * (51)	141 (54)	136 (58)	129 (60)	117 (64)
Cirrhosis-Alcohol	130 * (52)	131 * (53)	112 (59)	115 * (59)	97 (66)	91 (70)	90 (72)	74 (77)	89 (75)	68 (82)
Cirrhosis-Other	54 (78)	54 * (76)	40 (95)	43 (88)	29 (99)	29 (99)	25 (100)	20 (101)	28 (98)	17 (104)
PUD	47 (82)	36 (91)	61 (75)	37 (92)	83 (74)	48 (88)	110 (64)	67 (81)	144 (56)	90 (72)
Gastritis	5 * (120)	11 ## (110)	1 (130)	3 * (124)	9 (114)	0 (132)	12 ## (112)	9 (110)	20 (104)	11 ## (109)
Appendicitis	3 (126)	1 (132)	3 (125)	2 (128)	5 (122)	2 (126)	5 (120)	4 (121)	9 (116)	4 (121)
Ileus	27 ## (99)	19 ## (102)	42 * (90)	29 ## (98)	52 (88)	48 * (89)	81 * (79)	57 (84)	120 (63)	94 * (71)
I+F Hernia	18 (107)	15 (106)	22 (105)	22 (104)	25 (102)	26 (102)	30 (96)	29 (95)	35 (95)	33 (95)
IBD	32 ## (96)	36 (89)	28 * (102)	32 ## (95)	25 (103)	26 * (103)	26 (98)	22 (100)	32 (96)	24 (99)

Table S3. *Cont.*

Short Cause Names	Average DALYs per 100,000 (Rank in 136 Causes)									
	60–64 Years		65–69 Years		70–74 Years		75–79 Years		80–84 Years	
	2015	2020	2015	2020	2015	2020	2015	2020	2015	2020
Vasc Intest	26 (100)	28 (98)	52 (86)	37 (93)	89 (70)	83 (74)	122 (60)	141 (56)	153 (55)	180 (52)
Gall + Bile	64 (73)	52 (78)	93 (65)	71 (73)	130 (56)	110 (62)	162 (53)	158 (54)	210 (47)	194 (51)
Pancreatitis	35 (94)	33 (93)	41 (94)	39 (91)	50 (89)	46 (90)	64 (85)	55 (86)	77 (82)	73 (80)
Oth Diges	237 (34)	197 * (40)	304 (32)	270 (35)	394 (29)	352 (31)	470 (30)	463 (30)	629 (27)	539 (31)
Alzh	201 (38)	171 * (44)	310 (31)	353 (29)	528 (27)	502 (26)	1048 (16)	884 (20)	2082 (12)	1905 (15)
Parkins	38 * (89)	33 (95)	63 (74)	50 * (82)	123 (57)	89 (72)	213 (48)	194 (49)	286 (38)	346 (36)
Epilepsy	86 (67)	73 (70)	82 (71)	75 (71)	78 (77)	73 (77)	86 (73)	70 (80)	89 (73)	82 (76)
MS	19 # (106)	14 # (107)	14 * (107)	18 # (107)	12 * (112)	11 * (109)	4 (123)	9 * (108)	7 (118)	2 (127)
Migraine	171 (42)	198 # (38)	141 (51)	159 (49)	112 (61)	126 (57)	92 (71)	98 (68)	80 (80)	79 (78)
Headache	33 (95)	33 (94)	33 (99)	33 (94)	32 (97)	32 (97)	31 (95)	31 (94)	31 (97)	31 (96)
Oth Neuro	103 (62)	102 (63)	125 (56)	150 (54)	130 (55)	173 (50)	126 (58)	167 (53)	115 (65)	149 (58)
Schizo	420 * (26)	490 (23)	328 (27)	391 * (28)	246 (41)	287 (37)	186 (49)	203 (48)	144 (57)	148 (59)
Alcohol	314 # (29)	384 ## (28)	216 (40)	293 # (33)	135 (53)	182 (47)	81 (78)	104 (66)	53 (91)	58 (88)
Drugs	39 (88)	42 (84)	23 (104)	26 (103)	14 (107)	15 (106)	14 (108)	9 (112)	13 (112)	10 (110)
Unipolar	1155 (11)	1185 (11)	1066 # (14)	1165 (13)	928 (16)	1045 # (16)	824 (20)	875 (21)	865 * (20)	759 (23)
Bipolar	144 (50)	159 (46)	117 (58)	115 (60)	96 (68)	94 (67)	76 (82)	78 (76)	59 (89)	62 (87)
Anxiety	218 (36)	232 * (37)	201 (43)	209 (40)	184 (48)	190 (45)	170 (52)	172 (52)	160 (54)	158 (55)
Eat Disorder	5 (121)	5 (119)	4 (123)	4 (121)	3 (124)	3 (121)	3 (125)	3 (125)	5 ## (124)	3 (124)
PDD	101 (64)	103 (62)	94 (64)	97 (65)	88 (72)	90 (71)	81 (80)	84 (74)	71 (85)	76 (79)
Child Behav	0 (135)	0 (135)	0 (136)	0 (135)	0 (136)	0 (136)	− (−)	− (−)	− (−)	− (−)
Intellect	2 (130)	3 (124)	1 (133)	1 (130)	0 (134)	0 (133)	0 (133)	0 (133)	0 (133)	0 (133)
Oth Mental	23 * (102)	16 ## (105)	33 (98)	26 * (102)	44 (93)	39 (95)	54 (90)	54 (87)	76 (83)	65 (84)
Diabetes	1655 (8)	1653 (7)	2157 (8)	2144 (7)	2733 (7)	2764 (8)	3252 (5)	3447 (6)	3628 (6)	3995 (6)
Glom	8 (114)	5 (120)	9 (114)	6 (119)	13 (111)	6 (117)	17 (106)	9 (109)	19 (106)	13 (108)
CKD	474 (21)	394 (27)	661 (20)	569 (22)	827 (18)	822 (20)	958 (19)	1022 (17)	1125 (17)	1163 (18)

Table S3. *Cont.*

**Short Cause Names**	**Average DALYs per 100,000 (Rank in 136 Causes)**									
	**60–64 Years**		**65–69 Years**		**70–74 Years**		**75–79 Years**		**80–84 Years**	
	**2015**	**2020**	**2015**	**2020**	**2015**	**2020**	**2015**	**2020**	**2015**	**2020**
Urinary	659 (17)	746 (16)	724 (19)	1110 (14)	717 (22)	1032 (17)	676 (25)	886 (19)	617 (28)	754 (24)
Gynecol Dis	100 (65)	88 (65)	75 (72)	81 (69)	30 (98)	60 (79)	13 (111)	19 (102)	18 (108)	7 (114)
Hemog	90 # (66)	76 ## (69)	125 (55)	95 # (67)	141 (52)	142 (54)	180 (50)	157 (55)	260 (41)	205 (49)
Oth Endo	146 (49)	145 (50)	176 (45)	187 (43)	204 (44)	224 (41)	228 (42)	258 (42)	254 (42)	281 (40)
R Arthritis	132 (51)	115 (56)	159 (47)	158 (50)	192 (47)	188 (46)	222 (45)	225 (45)	282 (40)	259 (42)
Osteo	1288 (10)	2433 (6)	1138 (12)	1701 (10)	1055 (15)	1295 (13)	1042 (17)	1113 (16)	1056 (18)	1073 (20)
Low Back + Neck	2790 (4)	3019 (4)	2838 (5)	2986 (4)	2920 (6)	2985 (6)	3024 (8)	3051 (7)	3127 (7)	3152 (8)
Gout	5 (122)	6 (116)	5 (119)	6 (116)	6 (118)	6 (116)	6 (116)	7 (116)	6 (122)	7 (115)
Oth Musculo	760 (16)	572 ## (20)	1031 (15)	856 (17)	1243 (13)	1245 (14)	1236 (15)	1505 (14)	893 ## (19)	1404 (17)
Congenital	58 (75)	44(83)	56 (81)	56 (78)	50 (90)	55 (84)	43 (92)	48 (90)	38 (94)	41 (93)
Skin D	471 (22)	430 * (25)	529 (24)	494 (25)	572 (25)	565 (23)	630 (26)	609 (26)	727 (24)	672 (28)
Sense Org	920 (14)	798 (14)	1394 (10)	1171 (12)	2213 (9)	1836 (10)	3098 (6)	3036 (8)	3848 (5)	4183 (5)
Oral Cond	413 (27)	414 (26)	467 (25)	453 (26)	538 (26)	512 (25)	579 (28)	591 (28)	564 * (30)	622 (30)

* $0.35 < C \le 0.5$, the fitting effect is eligible; # $0.5 < C \le 0.65$, the fitting effect is below standard, but not the worst; ## $C > 0.65$, the fitting effect is fairly unsatisfactory. The average DALYs (per 100,000) are rounded to the nearest integers in the table, and the rank lists are got according to the original data. The full cause names are presented in Table S5.

Table S4. Disability adjusted life years (DALYs) for 12 injuries.

**Short Cause Names**	**Average DALYs per 100,000 (Rank in 136 Causes)**									
	**60–64 Years**		**65–69 Years**		**70–74 Years**		**75–79 Years**		**80–84 Years**	
	**2015**	**2020**	**2015**	**2020**	**2015**	**2020**	**2015**	**2020**	**2015**	**2020**
Road Inj	1098 * (12)	1138 (12)	952 (16)	1044 * (16)	891 * (17)	888 (19)	790 (21)	844 * (22)	710 (25)	748 (25)
Oth Trans	68 ## (71)	80 ## (66)	55 # (82)	65 ## (75)	47 (92)	51 # (87)	47 (91)	41 (93)	49 # (93)	43 (92)
Fall	1046 (13)	1028 (13)	1200 (11)	1247 (11)	1373 (11)	1421 (11)	1663 (12)	1612 (13)	2270 (11)	1968 (12)

Table S4. *Cont.*

**Short Cause Names**	**Average DALYs per 100,000 (Rank in 136 Causes)**									
	**60–64 Years**		**65–69 Years**		**70–74 Years**		**75–79 Years**		**80–84 Years**	
	**2015**	**2020**	**2015**	**2020**	**2015**	**2020**	**2015**	**2020**	**2015**	**2020**
Drown	116 (57)	91 (64)	124 (57)	102 (62)	122 (58)	113 (60)	136 (56)	110 (64)	171 # (53)	130(62)
Fire	45 (85)	41 (88)	46 * (88)	42 (89)	54 ## (86)	44 * (92)	78 # (81)	54 ## (88)	108 (69)	86 # (73)
Poison	120 (54)	114 * (58)	110 (60)	100 (63)	102 (64)	92 (69)	101 (68)	84 (72)	103 (70)	86 (74)
Mech Force	67 (72)	67 (72)	42 (92)	53 (81)	33 (95)	30 (98)	25 (101)	24 (99)	28 (100)	19 (102)
Med Treat	52 (80)	42 (86)	65 (73)	69 (74)	88 (71)	87 (73)	86 (74)	124 (62)	108 (68)	110 (65)
Animal	14 (110)	10 (112)	13 (110)	12 (109)	11 (113)	10 (111)	11 (113)	8 (113)	12 (113)	8 (112)
Oth Unintent	119 (55)	108 * (59)	86 * (67)	95 (66)	91 (69)	64 * (78)	96 * (70)	74 (78)	116 # (64)	83 * (75)
Self-harm	587 (19)	431 (24)	614 (21)	534 (23)	642 (23)	560 (24)	776 * (22)	596 (27)	820 ## (22)	764 * (22)
Violence	40 (87)	34 (92)	29 (101)	27 (100)	24 (104)	20 (104)	19 (105)	17 (106)	16 (109)	13 (107)

* 0.35 < C ≤ 0.5, the fitting effect is eligible. # 0.5 < C ≤ 0.65, the fitting effect is below standard, but not the worst. ## C > 0.65, the fitting effect is fairly unsatisfactory. The average DALYs (per 100,000) are rounded to the nearest integers in the table, and the rank lists are got according to the original data. The full cause names are presented in Table S5.

Table S5. The full names of 136 predicted causes.

Short Cause Names	Full Cause Names
AA	Aortic aneurysm
AFib	Atrial fibrillation and flutter
Alcohol	Alcohol use disorders
Alzh	Alzheimer's disease and other dementias
Animal	Animal contact
Anxiety	Anxiety disorders
Appendicitis	Appendicitis
Asthma	Asthma
Bipolar	Bipolar affective disorder
Bladder	Bladder cancer
Brain	Brain and nervous system cancers
Breast	Breast cancer
Cervix	Cervical cancer
Child Behav	Childhood behavioral disorders
Cirrhosis-Alcohol	Cirrhosis of the liver secondary to alcohol use
Cirrhosis-HepB	Cirrhosis of the liver secondary to hepatitis B
Cirrhosis-HepC	Cirrhosis of the liver secondary to hepatitis C
Cirrhosis-Other	Other cirrhosis of the liver
CKD	Chronic kidney diseases
CMP	Cardiomyopathy and myocarditis
Colorectal	Colon and rectum cancers
Congenital	Congenital anomalies
COPD	COPD
Cysticer	Cysticercosis
Dengue	Dengue
Diabetes	Diabetes mellitus
Diarrhea	Diarrheal diseases
Drown	Drowning
Drugs	Drug use disorders
Eat Disorder	Eating disorders
Echino	Echinococcosis
Enceph	Encephalitis
Endocarditis	Endocarditis
Epilepsy	Epilepsy
Esophagus	Esophageal cancer
Fall	Falls
FBT	Food-borne trematodiases
Fire	Fire, heat and hot substances
Gall + Bile	Gall bladder and bile duct disease
Gallbladder	Gallbladder and biliary tract cancer
Gastrititis	Gastritis and duodenitis
Glom	Acute glomerulonephritis
Gout	Gout
Gynecol Dis	Gynecological diseases

Table S5. *Cont.*

Short Cause Names	Full Cause Names
Headache	Tension-type headache
Short Cause Names	Full Cause Names
Hemog	Hemoglobinopathies and hemolytic anemias
Hep	Hepatitis
HIV	HIV/AIDS
Hodgkin's	Hodgkin's disease
HTN Heart	Hypertensive heart disease
I+F Hernia	Inguinal or femoral hernia
IBD	Non-infective inflammatory bowel disease
IHD	Ischemic heart disease
Ileus	Paralytic ileus and intestinal obstruction without hernia
Int Lung	Interstitial lung disease and pulmonary sarcoidosis
Intellect	Idiopathic intellectual disability
Iodine	Iodine deficiency
Iron	Iron-deficiency anemia
Kidney	Kidney and other urinary organ cancers
Larynx	Larynx cancer
Leish	Leishmaniasis
Leprosy	Leprosy
Leukemia	Leukemia
Liver	Liver cancer
Low Back + Neck	Low back and neck pain
LRI	Lower respiratory infections
Lung	Trachea, bronchus, and lung cancers
Lymphoma	Non-Hodgkin lymphoma
Malaria	Malaria
Mech Force	Exposure to mechanical forces
Med Treat	Adverse effects of medical treatment
Melanoma	Malignant melanoma of skin
Meningitis	Meningitis
Migraine	Migraine
Mouth	Mouth cancer
MS	Multiple sclerosis
Myeloma	Multiple myeloma
N Enceph	Neonatal encephalopathy (birth asphyxia and birth trauma)
Naso	Nasopharynx cancer
Nematode	Intestinal nematode infections
NMSC	Non-melanoma skin cancer
Oral Cond	Oral disorders
Osteo	Osteoarthritis
Oth Circ	Other cardiovascular and circulatory diseases
Oth Diges	Other digestive diseases
Oth Endo	Other endocrine, nutritional, blood, and immune disorders

Table S5. *Cont.*

Short Cause Names	Full Cause Names
Oth Inf	Other infectious diseases
Oth Mental	Other mental and behavioral disorders
Oth Musculo	Other musculoskeletal disorders
Oth Neoplasm	Other neoplasms
Oth Neuro	Other neurological disorders
Oth NTD	Other neglected tropical diseases
Oth Nutr	Other nutritional deficiencies
Oth Pharynx	Cancer of other part of pharynx and oropharynx
Oth Resp	Other chronic respiratory diseases
Oth Trans	Other transport injury
Oth Unintent	Unintentional injuries not classified elsewhere
Otitis	Otitis media
Ovary	Ovarian cancer
Pancreas	Pancreatic cancer
Pancreatitis	Pancreatitis
Parkins	Parkinson's disease
P-coniosis	Pneumoconiosis
PDD	Pervasive development disorders
PEM	Protein-energy malnutrition
Poison	Poisonings
Preterm	Preterm birth complications
Prostate	Prostate cancer
PUD	Peptic ulcer disease
PVD	Peripheral vascular disease
R Arthritis	Rheumatoid arthritis
Rabies	Rabies
Rheum HD	Rheumatic heart disease
Road Inj	Road injury
Schisto	Schistosomiasis
Schizo	Schizophrenia
Self-harm	Self-harm
Sense Org	Sense organ diseases
Skin D	Skin and subcutaneous diseases
STD	Sexually transmitted diseases excluding HIV
Stomach	Stomach cancer
Stroke	Cerebrovascular disease
TB	Tuberculosis
Testis	Testicular cancer
Tetanus	Tetanus
Thyroid	Thyroid cancer
Trachoma	Trachoma
Typhoid	Typhoid and paratyphoid fevers
Unipolar	Unipolar depressive disorders

Table S5. *Cont.*

Short Cause Names	Full Cause Names
URI	Upper respiratory infections
Urinary	Urinary diseases and male infertility
Uterus	Uterine cancer
Varicella	Varicella
Vasc Intest	Vascular disorders of intestine
Violence	Interpersonal violence
Vit A	Vitamin A deficiency

© 2015 by the authors; licensee MDPI, Basel, Switzerland. This article is an open access article distributed under the terms and conditions of the Creative Commons Attribution license (http://creativecommons.org/licenses/by/4.0/).
